# Oligo-Carrageenan Kappa Increases Expression of Genes Encoding Proteins Involved in Photosynthesis, C, N, and S Assimilation, and Growth in *Arabidopsis thaliana*

**DOI:** 10.3390/ijms241511894

**Published:** 2023-07-25

**Authors:** Tamara Méndez, Alejandra Fuentes, Diego Cofre, Alejandra Moenne, Daniel Laporte

**Affiliations:** 1 Laboratory of Plant Physiology and Molecular Biology, Instituto de Ciencias Biomédicas, Universidad Autónoma de Chile, Talca 3467987, Chile; tamendez@utalca.cl (T.M.); adfuente@uc.cl (A.F.); diego.cofre@alu.ucm.cl (D.C.); 2 Laboratory of Marine Biotechnology, Faculty of Chemistry and Biology, University of Santiago of Chile, Santiago 9170022, Chile

**Keywords:** *Arabidopsis thaliana*, basal metabolism, growth, oligo-carrageenan, photosynthesis, transcriptomic analyses

## Abstract

To analyze the effect of oligo-carrageenan (OC) kappa in the stimulation of growth in *Arabidopsis thaliana*, plants were sprayed on leaves with an aqueous solution of OC kappa at 1 mg mL^−1^, 5 times every 2 days and cultivated for 5 or 15 additional days. Plants treated with OC kappa showed an increase in rosette diameter, fresh and dry weight, and primary root length. Plants treated with OC kappa once and cultivated for 0 to 24 h after treatment were subjected to transcriptomic analyses to identify differentially expressed genes, mainly at 12 h after treatment. Transcripts encoding proteins involved in growth and development and photosynthesis were upregulated as well as enzymes involved in primary metabolism. In addition, plants treated with OC kappa once and cultivated for 0 to 96 h showed increased levels of transcripts encoding enzymes involved in C, N, and S assimilation at 6 and 12 h after treatment that remain increased until 96 h. Therefore, OC kappa increased the expression of genes encoding proteins involved in photosynthesis, C, N, and S assimilation, and growth in *A. thaliana*.

## 1. Introduction

The increase in world population and the decrease of cultivable land, due to desertification induced by climate change, are forcing humanity to find solutions to improve agricultural and forestry productivity to ensure food and housing for the population. In addition, the use of agrochemical fertilizers and pesticides has resulted in decreased soil fertility, loss of biodiversity and problems for human and animal health [1,2]. The stimulation of growth and productivity requires an increase in photosynthesis (carbon fixation from the air) as well as enhanced assimilation of nitrogen (N), sulfur (S), and phosphorus (P) from the soil because these light-dependent processes need to be coordinated to ensure proper growth and development of plants [3,4,5]. Thus, the development and implementation of new technologies that allow an increase in photosynthesis leading to an increase in productivity that is friendly with the environment is essential for sustainable development of the world.

In plants, growth is regulated by hormones such as auxins, gibberellins, cytokinins, and brassinosteroids. Auxins control the development of plant organs such as leaves, roots, and flowers, cell division in addition to expansion, phototropism of the shoots, gravitropism of the roots, and photosynthesis [6]. Cytokinins regulate cell division, development, function of chloroplasts, and inhibition of senescence [7]. Gibberellins are involved in stem elongation, germination, dormancy, flowering, and senescence [8]. Brassinosteroids control activation of the cell cycle, cell division and expansion, root and shoot growth, pollen germination, xylem formation and differentiation, and stimulation of senescence [9]. In this sense, tomato plants in the field treated with 20 ppm of auxin 4-chloro phenoxy-acetic acid, 20 ppm of gibberellin A3 (GA), and the mixture of both hormones each at 20 ppm showed an increase in yield of 31%, 38%, and 63% of tomato per hectare [10]. Thus, the use of plant hormones at low concentrations may be helpful to improve sustainable agriculture.

On the other hand, oligosaccharides derived from marine algae can stimulate plant growth [11,12,13]. Carrageenans are polysaccharides present in the cell wall of marine red algae, and they are constituted by galactose residues sulfated in positions 2, 4, and 6 and anhydrogalactose residues [14]. Carrageenans can be depolymerized to obtain oligo-carrageenans (OCs) by γ-irradiation or acid hydrolysis. In this sense, OC kappa obtained using γ-irradiation at a dose rate of 250 kGy h^−1^ resulted in a fraction of 20 kDa [15]. Peppermint plants treated with OC kappa at concentrations ranging from 0.04 to 0.2 mg mL^−1^, 5 times in total, and cultivated for 150 days showed that a concentration of 0.08 mg mL^−1^ induced the highest increase in rubisco activity (65.9%), an increase in phenylalanine ammonia lyase (PAL) activity of 35.6%, and an increase in the essential oil content of 32.8%, compared with control plants [15]. Interestingly, the non-depolymerized kappa carrageenan had no effect on peppermint plants [15]. Moreover, carrageenan kappa treated with 0.4 M of ascorbic acid and 2% of H_2_O_2_ at 90 °C for 60, 90, and 120 min produced OC kappa fractions of 42, 17, and 4 kDa, respectively [16]. Maize plants (*Zea mays* L.) treated with these OC kappa fractions at a concentration of 0.1 mg mL^−1^ 3 times in total and cultivated for 15, 30, and 50 days showed that an OC fraction of 4 kDa induced the highest increase in plant height of 6.9%, 15.4%, and 19.9% after 25, 40, and 60 days, respectively [16]. In addition, OC kappa induced an increase in N assimilation of 27.7%, 18.4% and 10.3% in leaves, trunk and grain, respectively, and an increase in P content of 15.38, 27.7% and 11.0%, respectively [16]. Thus, OC kappa fractions obtained by treatment with γ-irradiation or acid hydrolysis induced a stimulation of plant growth.

Furthermore, OCs kappa, lambda, and iota obtained by acid hydrolysis showed a molecular weight of around 10 kDa [14,17]. Tobacco plants (var. Xanthi) with OC kappa, lambda, and iota sprayed on leaves at a concentration of 1 mg mL^−1^ once a week, four times in total, and cultivated for two additional months showed an increase in fresh leaf biomass of 108%, 31%, and 121%, respectively [17]. Thus, mainly OCs kappa and iota stimulate growth in tobacco plants [17]. In addition, commercial tobacco plants (var. Burley) treated with OCs kappa, lambda, and iota showed an increase in leaf biomass of 131%, 126%, and 150%, respectively, indicating that OC iota is the most effective [18]. It was determined that tobacco plants (var. Burley) showed an increase in net photosynthesis, basal metabolism, and cell division [18]. In addition, tobacco plants showed a stimulation of basal metabolism due to an increase in activity of the enzyme ribulose 1,5-biphosphate carboxylase/oxygenase (rubisco), involved in C assimilation, and in activities of enzymes involved in purine and pyrimidine synthesis and fatty acid synthesis [18].

Furthermore, *Eucalyptus globulus* trees treated with OC kappa showed an increase in net photosynthesis and in the level of NADPH, which activates the thioredoxin reductases/thioredoxin system (TRR/TRX), which, in turn, increases C, N, and S assimilation by enhancing activities of rubisco as well as glutamine synthase (GlnS), involved in N assimilation, and O-acetylserine (thiol) lyase (O-ASTL), involved in S assimilation [19]. Interestingly, treatment with OC kappa increased the level of the auxin indole-acetic acid, gibberellin A3, and cytokine trans-zeatin, but decreased the level of the brassinosteroid epibrassinolide in *E. globulus* [20]. Thus, the increase in phytohormone level and in C, N, and S assimilation determined the enhanced growth observed in *Eucalyptus* treated with OC kappa.

In order to analyze the effect of OC kappa on *A. thaliana* growth, plants were sprayed on leaves with OC kappa at 1 mg mL^−1^ 5 times every 2 days and cultivated for 5 or 15 additional days, and different phenotypic parameters related to growth were analyzed. In addition, transcriptomic analyses were performed in plants treated once with OC kappa and cultivated for an additional 0 to 24 h. Transcripts of genes showing differential increased expression were selected, and those encoding proteins involved in photosynthesis and in C, N, and S assimilation were selected. In addition, the levels of chlorophylls a and b and carotenoids were quantified, and the increases in expression of genes involved in C, N, and S assimilation were corroborated by qRT-PCR.

## 2. Results

### 2.1. OC Kappa Induces an Increase in A. thaliana Growth

**Figure 1:**
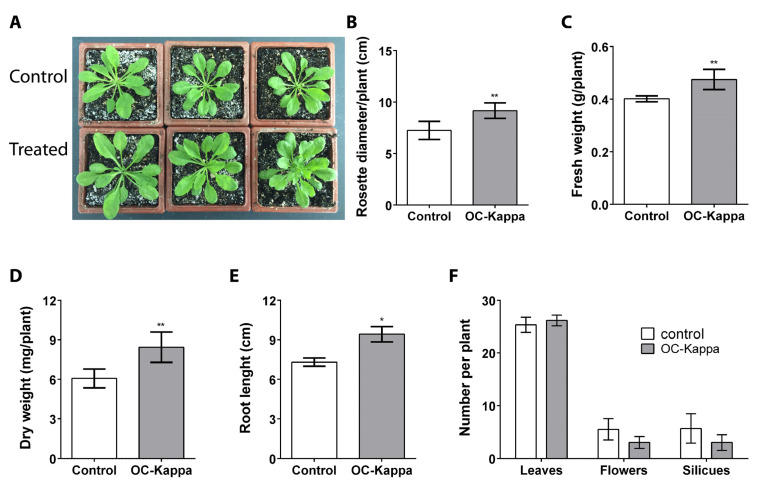
Rosettes of *A. thaliana* plants treated without OC kappa (controls, upper row) or with OC kappa 1 mg mL^−1^ 5 times every 2 days and cultivated for 5 additional days ((**A**), lower row). Increase in rosette diameter (**B**), fresh weight (**C**), dry weight (**D**), and root length (**E**) expressed as centimeters, grams per plant, milligram per plant, and centimeters, respectively, and number of leaves, flowers, and siliques (**F**) in control plants (open bars). Treated plants (black bars). Bars represent mean values of three independent experiments performed in triplicate ± SD. One asterisk represent significant differences among control and treated plants (* *p* < 0.05) and two asterisks represent significant differences (** *p* < 0.01).

To determine whether OC kappa can induce an increase in growth in *A. thaliana*, plants cultivated for 15 days were treated on leaves with an aqueous solution of OC kappa at a concentration of 1 mg mL^−1^ or with water (control) 5 times every 2 days and cultivated for 5 additional days (Figure 1A). The rosettes of control plants displayed an average diameter of 7.2 cm, whereas treated plants showed a diameter of 9.2 cm, which represents an increase of 15.5% in rosette diameter (Figure 1B). Control plants showed a fresh weight (FW) average of 0.4 g per plant, whereas treated plants showed an average FW of 0.47 g per plant, which represents an increase of 15.5% in FW (Figure 1C). Similarly, control plants showed an average dry weight (DW) of 6.1 mg per plant, and treated plants displayed 8.4 mg of DW per plant, which represents an increase of 28.1% in DW (Figure 1D).

In plants treated as described above but cultivated for 15 additional days, the length of the main root in control plants was 7.3 cm and in the treated plants was 9.3 cm, which represents an increase of 25.5% in root length (Figure 1E). In contrast, the number of leaves, flowers, and siliques in control and treated plants did not change, showing an average of 25 to 26 leaves, 3 to 5 flowers, and 3 to 5 siliques per plant (Figure 1F). Thus, OC kappa at a concentration of 1 mg mL^−1^ induced an increase in *A. thaliana* growth, reflected by the increase in rosette diameter, FW and DW of plants, and in the length of the primary root.

### 2.2. Identification of Transcripts Differentially Expressed in Response to OC Kappa

Plants were treated without OC kappa (controls) or with OC kappa at 1 mg mL^−1^ once, and samples were obtained from control and treated plants at 0, 6, 12, and 24 h after treatment. Transcriptomic analyses revealed 22,207 expressed genes and only 2 differentially expressed (0.001%) after 6 h; one was up-regulated and the other was down-regulated (Supplementary Figure S1A). After 12 h of treatment, 1821 genes (8.2%) were differentially expressed, 1257 genes were up-regulated (5.7%), and 564 were down-regulated (2.5%) (Supplementary Figure S1B). After 24 h of treatment, 1174 genes were differentially expressed (5.2%), 453 genes were up-regulated (2%), and 723 genes were down-regulated (3.2%) (Supplementary Figure S1C). Thus, treatment with OC kappa induced a differential expression of genes in *A. thaliana*, mainly after 12 h of treatment.

### 2.3. Classification of Differentially Expressed Transcripts in Response to OC Kappa

**Figure 2:**
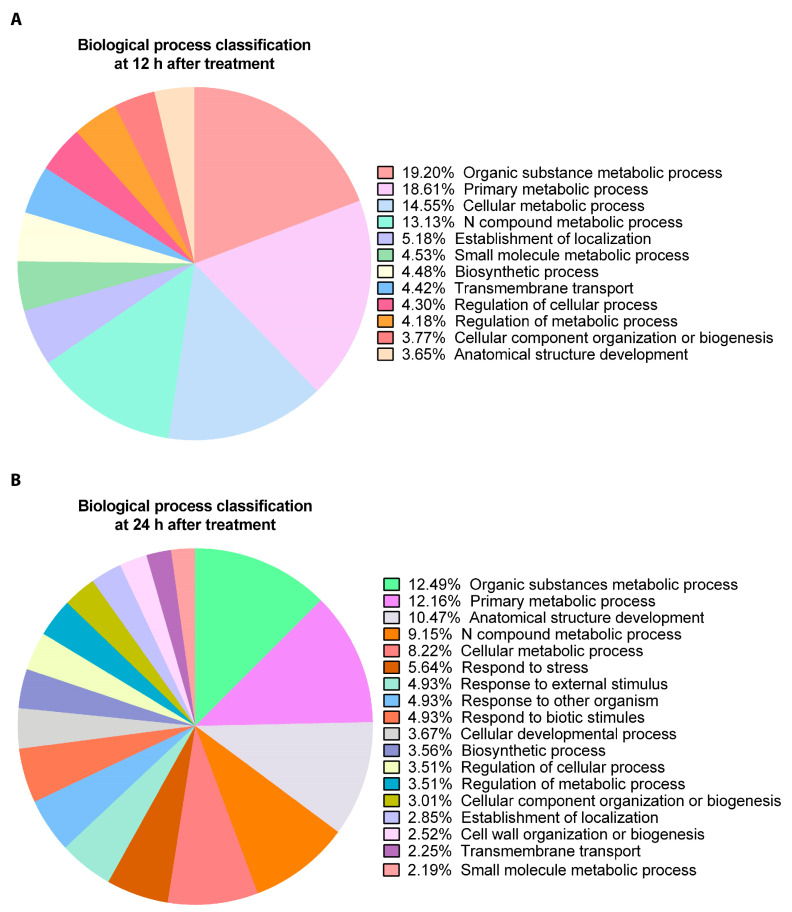
Pie chart of percentages of protein sequences associated with different biological processes of up-regulated transcripts in *A. thaliana* plants treated with OC kappa at 1 mg mL^−1^ once and cultivated for 12 h (**A**) and 24 h (**B**) after treatment.

Differentially expressed genes identified at 12 h after treatment were classified according to Gene Ontology (GO) into the following biological processes: 326 (19.20%) are involved in metabolic processes of organic substances; 316 (18.61%) in primary metabolic processes; 274 (14.55%) in cellular metabolic processes; 223 (13.13%) in metabolic processes of nitrogenous compounds; 88 (5.18%) in location establishment; 77 (4.53%) in metabolic processes of small molecules; 76 (4.48%) in biosynthetic processes; 75 (4.42%) in trans-membrane transport; 73 (4.3%) in regulation of cellular processes; 71 (4.18%) in regulation of metabolic processes; 64 (3.77%) in organization of cellular components or biogenesis, 62 (3.65%) in development of anatomical structures; and 68 genes (4%) in signaling processes (Figure 2A; Supplementary Table S2). Differentially expressed genes identified at 24 after treatment were classified into the following biological processes: 228 genes (12.49%) are involved in metabolic processes of organic substances; 222 (12.17%) in primary metabolic processes; 191 (10.47%) in development of anatomical structures; 167 (9.15%) in metabolic processes of nitrogenous compounds; 150 (8.22%) in cellular metabolic processes; 103 (5.64%) in stress response; 90 (4.93%) in response to external stimuli; 90 (4.93%) in response to biotic stimuli; 90 (4.93%) in response to another organism; 67 (3.67%) in cell development processes; 65 (3.56%) in biosynthetic processes; 64 (3.51%) in regulation of cellular processes; 64 (3.51%) in regulation of metabolic processes; 55 (3.01%) in organization of cellular components or biogenesis; 52 (2.85%) in establishment of location; 46 (2.52%) in organization of the cell wall and biogenesis; 41 (2.25%) in trans-membrane transport; 40 (2.19%) in metabolic processes of small molecules; and 45 (2.46%) in signaling processes (Figure 2B; Supplementary Table S2). Thus, after 12 h of treatment, around 82% of the differentially expressed genes are classified into cellular processes involved in primary metabolism and growth and development and 4% are related to signaling and regulation. In contrast, after 24 h of treatment, around 65.7% of differentially expressed genes are involved in cellular processes related to primary metabolism and growth and development and 2.46% are related to signaling and regulation, suggesting that the stimulation of growth and development is triggered as early as 12 h after treatment.

### 2.4. Identification of Transcripts Encoding Proteins Involved in Plant Growth and Development

There were 71 (4.2%) up-regulated genes identified at 12 h related to growth and development and associated with biological processes involved in plant growth, cell growth, pollen tube and meristem growth, seed germination, and plant growth and development by Gene Ontology (GO). The increased transcripts encoding proteins involved in plant growth were MOK16.3, a transporter involved in plant growth; MPN 9.9, a protein involved in phototrophic response; cytb451, a cytochrome located in the inner membrane of mitochondria and thylakoids; PECL, a pectin lyase; MMG15.21, a leucine-rich receptor; and ACT, an acyl transferase (Table 1). In addition, increased transcripts encoding proteins involved in cell growth were AOP1, a 2-oxoglutarate-dependent dioxygenase; UBQT, a ubiquitin transferase; DTX27, a detoxification antiporter; HAD, an acid phosphatase; EXPA1, EXP3, and EXP8, three expansins involved in cell growth; GASA1, a protein involved in seed germination dependent on gibberellins; and HBI1, a bHLH transcription factor (Table 1). The increased transcripts encoding proteins involved in pollen tube and meristem growth and modification of the cell wall were OASB, a chloroplastic cysteine synthase; NAS1 and NAS3, two nicotianamine synthases that produce nicotianamine involved in iron and zinc transport and accumulation; TOD, a ceramidase; WD-40, a protein involved in signal transduction; GASA6, a gibberellin-dependent protein involved in seed germination; and PGLR4, a polygalacturonase (Table 1). Moreover, the increased transcripts encoding proteins involved in developmental growth were TES, a thioesterase; SurE, a nucleotidase; HAD, a phosphatase; SAUR29, an auxin-responsive protein; BAM9, a beta-amylase; ACR1, an actin-domain-containing protein; RXW8 and GDSL, two lipases; HIPPO9, an ABC transporter; MSL1.19, an asparraginase; LRX4, an extensin-like protein; YSL6, an iron-nicotinamide transporter; PLY20, a pectate lyase; BIG1E, a big grain protein; DVL6, a rotundifolia-like protein; T32F6.3, a TPR-containing protein; CBEP, a calcium-binding endonuclease; DL3410C, an auxin-dependent protein; BGAL8, a beta-galactosidase; and BHLH149, a bHLH-type transcription factor involved in brassinosteroids signaling (Table 1). Finally, increased transcripts encoding for proteins involved in the regulation of plant growth and development were PRE1 and PRE6, two transcription factors implicated in gibberellin-dependent response and light signal transduction, respectively; PILS5, an auxin transporter; HO1, a chloroplast heme oxygenase; CPK11, a calcium-dependent protein kinase; GLV2, a signaling protein involved in root growth; SAUR78, an auxin-responsive protein; EBP1, a protein that binds to mature rRNAs; CBD; CAP, a calmodulin-binding protein; and GSTU17, a protein involved in defense against pathogens (Table 1). Thus, OC kappa induced an increase in the level of transcripts encoding proteins such as auxin-, gibberellin-, and brassinoisteroids-responsive proteins involved in growth and development; extensins, polygalacturonases, galactosidases, and pectin lyase, involved in cell growth and expansion; and several transcription factors, among others.

### 2.5. Identification of Transcripts Encoding Proteins Involved in Photosynthesis

Since OC kappa induces an increase in the expression of genes related to growth and development, transcripts encoding proteins involved in photosynthesis were analyzed using transcriptomic analyses. Increased transcripts that encode proteins of photosystem II (PSII) were Psb27, Psb28, and Psb29, involved in the biogenesis of photosystem II (PSII), and subunits of PSII, including PsbK, PsbO1, PsbO2, PsbP1, PsbQ1, PsbQ2, PsbR PsbS PsbT, PsbW, PsbX, and PsbY. Those of photosystem I (PSI) were PsaD1, PsaE1, PsaE2, PsaF, PsaG, PsaH, PsaK, PsaL, PsaN, PsaO, and PGRL1A, a ferredoxin-plastoquinone reductase (Table 2). Increased transcripts encoding proteins of the Light Harvesting Complex II (LHCII) were LhcB 1.1, LhcB 1.2, LhcB 1.3, LhcB 1.5, LhcB 2.1, LhcB 2.2, LhcB 2.4, LhcB 3, LhcB 4.1, LhcB 4.2, LhcB 5, LhcB 6, and LhcB 7, and those encoding subunits of Light Harvesting Complex I (LHCI) were LHCA1, LHCA2, LHCA3, LHCA4, LHCA5, LHCA6, and LIL3.1, a protein of the LHCI-like complex (Table 2). Increased transcripts encoding proteins involved in electron transport, chlorophyll synthesis, repair and reassembly of PSII, and in ATP synthase activity were PNSB1, photosynthetic NADPH dehydrogenase subunit 48; PETE, plastocyanin; PPP1, potential lipid transporter; CHLP, geranyl diphosphate reductase; CRD1, magnesium protoporphyrin IX cyclase involved in chlorophyll synthesis; ELIP2, early light-induced protein 2; Hcf173 and Hcf244, two HIGH CHLOROPHYLL FLUORESCENCE PHENOTYPE proteins; Lqy1, a protein disulphide isomerase; OHP1, LHC-like protein involved in repair and reassembly of PSII; and ATPaseF1, subunit delta of ATP synthase F1 complex (Table 2). Thus, the levels of transcripts encoding proteins belonging PSII, LHCII, PSI, LHCI, and repair of PSII were increased in response to OC kappa, suggesting that an increase in photosynthesis may participate in the stimulation of growth in *A. thaliana* plants.

### 2.6. Quantification of Chlorophylls and Carotenoids in Plants Treated with OC Kappa

**Figure 3:**
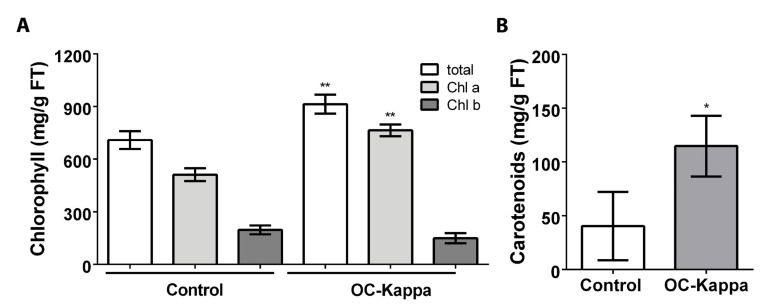
Level of total chlorophyll (chl), chl a and chl b, (**A**) and carotenoids (**B**) in *A. thaliana* plants without treatment (control) and treated with OC kappa at 1 mg mL^−1^ five times in total every two days and cultivated for five additional days. The level of chlorophylls is expressed as micrograms per gram of fresh weight. Bars represent mean values of three independent experiments performed in triplicate ± SD. One asterisk represent significant differences among control and treated plants (* *p* < 0.05) and two asterisks represent significant differences (** *p* < 0.01).

To analyze whether photosynthesis may be increased in *A. thaliana* plants treated with OC kappa, the levels of chlorophyll (chl) a and b and the level of carotenoids were determined. The level of chl a in control plants was 511.9 µg g^−1^ of FT, and in treated plants it was 764.4 µg g^−1^ of FT, which corresponds to an increase of 1.5 times in chl a level (Figure 3A). The level of chl b in control plants was 196.7 µg g^−1^ of FT, and in treated plants it was 149.0 µg g^−1^ of FT, but these levels were not significantly different (Figure 3A). Total chlorophyll level in control plant was 708.6 µg g^−1^ of FT, and in treated plants it was 913.4 µg g^−1^ of FT, which corresponds to an increase of 1.3 times (Figure 3A). The level of carotenoids in control plants was 40.3 µg g^−1^ of FT, and in treated plants it was 114.7 µg g^−1^ of FT, which represents an increase of 2.8 times (Figure 3B). Thus, treatment with OC kappa increased in the levels of total chlorophyll and carotenoids, suggesting that photosynthesis may be enhanced in *A. thaliana* plants.

### 2.7. Identification of Transcripts Encoding Enzymes Involved in C, N, and S Assimilation

To analyze whether OC kappa induces changes in the transcripts encoding proteins related to C, N, and S assimilation, control and treated plants were analyzed using transcriptomic analyses. Increased transcripts encoding enzymes related to Calvin–Benson cycle were FD1 and FDC1, two ferredoxin 1; FNR1, ferredoxin NADP reductase isoenzyme 1; FBA2 and FBA1, two isoenzymes of fructose biphosphate aldolase; G3PDHA2, G3PDHB, and G3PDHA1, three isoforms of glyceraldehyde 3-phophate dehydrogenase; PGRL1A, ferredoxin-plastoquinone reductase; R5PI2 and R5PI3, two isoforms of ribose 5-phosphate isomerase; RBCS-1A, RBCS-1B, RBCS-3B, and RBCS-2B, four isoforms of rubisco small subunit; RBCA, rubisco activase involved in the stimulation of rubisco activity; RAF1.2, rubisco accumulation factor; RBCX2, chaperonin; TRXF1, TRXF2, and TRXM1, three thioredoxins; ClTRX and CDSP32, two thioredoxin-like proteins; XPT, xylulose 5-phosphate translocator; TPT, triose phosphate translocator; and PPT2, a phosphoenol pyruvate translocator (Table 3). Moreover, increased transcripts encoding proteins and enzymes involved nitrogen assimilation were GATA21, a transcription factor; GLN1-2, cytosolic glutamine synthase; GLN2, chloroplastic glutamine synthase; NPF1.2, NPF6.2, NPF6.3, and NPF6.4, four nitrate transporters; NIR1, a nitrite reductase; and chloroplastic ferredoxin nitrate reductase (Table 3). Furthermore, the level of transcripts encoding proteins and enzymes involved in sulfur assimilation were APR2 and APR3, two isoforms of chloroplastic 5′-adenylylsulfate reductase; APK4, an adenylyl sulfate kinase; OASB, DES1, and CYSD2, three isoforms of chloroplastic cysteine synthase; SULTR2 and SULTR3, two sulphate transporters, and two TAUE2, two isoforms of sulfide exporter (Table 3). Thus, OC kappa increased the level transcripts encoding proteins and enzymes involved in C, N, and S assimilation that may participate in the stimulation of growth in *A. thaliana* plants.

### 2.8. Quantification of Transcripts Encoding Enzymes Involved in C, N, and S Assimilation

**Figure 4:**
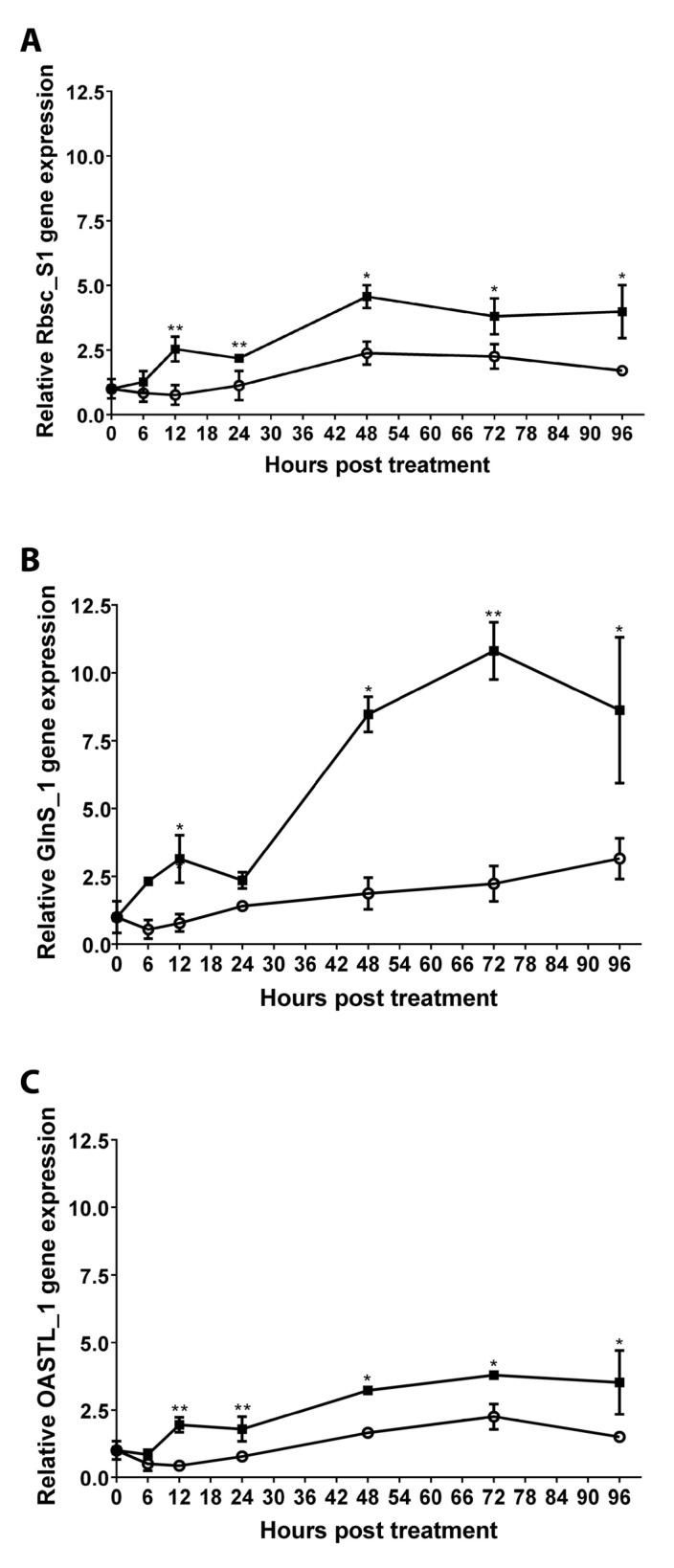
Relative level of transcripts encoding rubisco small subunit S1 (**A**), glutamine synthase 1 (**B**), and O-acetyl thiol lyase 1 (**C**) in control *A. thaliana* (open circles) and in plants treated with OC kappa at 1 mg mL^−1^ (black squares), once, and cultivated for 0 to 96 h after treatment. The relative level of transcripts is expressed as 2^−∆∆CT^. Symbols represent mean values of three independent experiments performed in triplicate ± SD. One asterisk represent significant differences among control and treated plants (* *p* < 0.05) and two asterisks represent significant differences (** *p* < 0.01).

To determine the temporal pattern in the increase of transcripts encoding an enzyme related to carbon fixation, Rbc_S1, an enzyme involved in N assimilation, GlnS_1, and an enzyme involved in S assimilation, OASTL_1, their relative levels were determined in control and treated plants at 0, 6, 12, 24, 48, 72, and 96 h after a single treatment. The level of transcripts encoding Rbsc_S1 increased by 3.34 times at the 12 h after treatment and remained increased until 96 h by 2.34 times compared to control plants (Figure 4A). The levels of GlnS_1 transcript increased by 4.22 times at 6 after treatment and remained increased by 4.85 times until 96 h after treatment compared to control plants (Figure 4B). The level of transcript encoding OASTL_1 increased by 4.48 times at 12 h after treatment and remained increase by 2.35 times at 96 h after treatment (Figure 4C). Thus, *A. thaliana* plants treated with OC kappa showed increases in the level of transcripts encoding enzymes involved in C, N, and S assimilation beginning at 6–12 h after treatment and continuing until 96 h after treatment.

## 3. Discussion

In this work, we showed that OC kappa stimulates growth in *A. thaliana* plants by increasing FW by 15.5% and DW by 28.1%, indicating that the increase in DW is higher than in FW. This result indicates that treated plants may have a higher content of biological macromolecules, and not a higher content of water, indicating that OC kappa induced the synthesis of biological macromolecules in *A. thaliana*. Moreover, this result is in accord with previous results observed in laboratory tobacco plants (var. Xhanti) treated with 1 mg mL^−1^ of OC kappa that displayed an increase in FW of 108% [17] and in field tobacco plants (var. Burley) showing an increase in FW of 1.9 times [18]. A similar effect was observed in peppermint sprayed with an aqueous solution of OC kappa at 0.08 mg mL^−1^ that showed an increase in FW of 46.9% and in DW of 53.5% [15]. Thus, OC kappa induced a higher increase in DW in different plants, indicating that a higher content of biological macromolecules may be synthesized. In contrast, treatment with OC kappa did not change the number of leaves, flowers, or siliques. Similarly, tobacco plants treated with OC kappa at 1 mg mL^−1^ did not show differences in the number of leaves but displayed an increase in height by 200% [17]. In addition, San et al. (2020) [16] showed that OC kappa of 4 kDa showed an increase in height of 6.9–19.9% in maize plants, indicating that OC kappa can induce increased growth in monocots and dicots.

An enhanced growth of *A. thaliana* plants was associated with transcriptomic analyses showing an increase in the expression of several genes involved in plant growth and development. Among up-regulated genes, auxin- and gibberellin-responsive proteins and a bHLH transcription factor involved in brassinosteroids signaling were found. Thus, it is possible that treatment of *A. thaliana* plants with OC kappa may increase the level of auxin, gibberelins, and brassinosteroids in *A. thaliana* plants. In this sense, it has been shown that the auxin 3-indole acetic acid, gibberellin A3, and the cytokinin trans-zeatin levels were increased in *Eucalyptus globulus* trees treated with OC kappa, but the level of epibrassinolide was decreased [20]. On the other hand, *A. thaliana* plants treated once with OC kappa and harvested at 24 h after treatment showed a decrease in the level of transcripts encoding two DELLA proteins and receptor-like kinase MOL1 (Supplementary Table S3). DELLA proteins are involved in repression of gibberellins synthesis and inhibit cell proliferation and expansion, and, thus, inhibit growth [21,22]. MOL1 is an inhibitor of cambium cell activity, leading to a decrease in secondary growth of the cell wall [23,24]. Thus, the inhibition in expression of these inhibitory regulators may also lead to an increase in growth of *A. thaliana* plants. Thus, OC kappa may increase the level of plant hormones involved in the stimulation of growth and down-regulate transcripts that encode inhibitors of growth in *A. thaliana*.

On the other hand, plant cell wall extension is the consequence of a turgor-driven extension of the primary cell wall. Transcriptomic analyses showed an increase in the level of transcripts encoding extensins, polygalaturonases, galactosidases, and pectin lyases in response to OC kappa. Extensins, arabinogalactan proteins, and pectin lyase participate in the formation of primary cell wall, and glucuronidases and galactosidases are required for cell wall loosening and cell expansion. Extensins are basic proteins with a high content of lysines and hydroxyproline that can be cross-linked with each other at tyrosine residues to form intra-molecular bridges and a molecular network in plant cell walls [25]. Extensins can bind to pectin and to arabinogalactan proteins, and they are glycosylated proteins that increase firmness of the cell wall and the cessation of growth of the primary cell wall [26]. On the other hand, cellulose and xyloglucans, but also pectin, are the main components of the cell wall. Pectin is synthesized by pectin lyases, and pectin is constituted by rhamnogalacturonans and xylogalacturonans [27]. Polygalacturonases are involved in pectin degradation and in cell loosening and expansion [28]. In addition, arabinogalactan proteins are highly glycosylated in arabinose and galactose, and they are also cross-linked with each other, and galactosidases are also involved in cell wall loosening and cell expansion [29]. Moreover, the levels of a xyloglucan hydrolase were increased in plants treated with OC kappa, once, and analyzed 12 h after treatment (Supplementary Table S3). Xyloglucan hydrolase is involved in primary cell wall degradation and thus increases growth [30]. Thus, OC kappa induced an increase in the expression of genes involved in cell wall loosening, cell expansion, and growth in *A. thaliana*.

Transcriptomic analyses showed an increase in the expression of genes encoding subunits of LHCII, PSII, LHCI, and PSI as well as in proteins involved in repair and reassembly of PSII. In addition, OC kappa induced an increase in the levels of chl a and carotenoids but not in chl b. It has been shown that the level of total chlorophyll is correlated with an increase in net photosynthesis [31]. The increase in total chlorophyll induced by OC kappa suggests that photosynthesis may be increased in *A. thaliana*. In addition, the increase in carotenoids level in response to OC kappa may protect PSII and PSI from oxidative stress induced by the potential increase in net photosynthesis. In fact, net photosynthesis was increased in tobacco plants treated with OC kappa, as well as the level of chl a and chl b [18]. In addition, net photosynthesis was also increased in *E. globulus* trees treated with OC kappa [19]. Thus, it is possible that net photosynthesis is increased in *A. thaliana* plants treated with OC kappa.

These analyses also indicate that the levels of transcripts encoding enzymes involved in C, N, and S assimilation are increased, suggesting that basal metabolism is stimulated in response to OC kappa in *A. thaliana* plants. The latter is in accord with results obtained in tobacco plants treated with OC kappa that showed an increase in C, N, and S assimilation as well as in *E. globulus* trees treated with OC kappa [18,20]. Furthermore, banana plants treated with carrageenan lambda showed an increase in N uptake [32], and peppermint plants treated with oligo-carrageenan kappa showed a higher accumulation of N in leaves [15]. In addition, the increase in the level of transcripts encoding enzymes involved in C, N, and S assimilation was verified by qRT-PCR in *A. thaliana* plants. These transcripts showed a sustained increase at 6 to 12 h after treatment that remained until 96 h after treatment. On another hand, banana plants treated with carrageenan lambda showed an increase in the level of transcripts encoding the large subunit of rubisco (rbcL) by 8.1 times, which suggests an increase in carbon assimilation [32]. In this sense, tobacco plants treated with OC kappa showed an increase in the activity of rubisco, but the amount of rubisco larger subunit was similar in control and treated plants, suggesting that activity of rubisco activase (RA) may be increased [18]. Interestingly, *A. thaliana* plants treated with OC kappa showed an increase in the level of transcripts encoding RA as well as the level of transcripts encoding four isoforms of the small subunit of rubisco. Thus, it is possible that in *A. thaliana,* the activity of rubisco enzyme may also be increased. It has been shown that the increase in photosynthesis may induce an increase in NADPH level and the activation of the thioredoxin (TRX)/thioredoxin reductase (TRXR) system that regulates the activity of RA and 4 enzymes of the 11 enzymes of the Calvin–Benson cycle, including glyceraldehyde 3-P dehydrogenase (G3PDH) [33]. Here, it was shown that the expression of three TRX F and M that control activities of several enzymes of the Calvin–Benson cycle was increased as well as three isoforms of G3PDH and RA [34]. Thus, activities of G3PDH and RA may be controlled by transcriptional regulation as well as post-transcriptional regulation through the TRX/TRXR system, including TRX F and M.

## 4. Materials and Methods

### 4.1. Plant Cultivation and Preparation of OC Kappa

Seeds of *A. thaliana* ecotype Columbia (col-0) were sterilized using 50% sodium hypochlorite solution, stratified at 4 °C for 48 h, germinated and grown in a mixture of compost/vermiculite (3:2) with 12 h light, 100 µmol m^−2^ s^−1^, at 22 ± 2 °C in a growth chamber (Velp, Usmate Velate, Italy) for 15 days and then subjected to treatments.

Carrageenan kappa (20 g) was purchased from Gelymar S.A. (Santiago, Chile) and solubilized in 2 L of distilled water at 60 °C, concentrated HCl (36.2 N) was added to reach a concentration of 0.1 M, then the solution was incubated for 45 min and neutralized to pH = 7 by addition of 0.1 N NaOH.

### 4.2. Treatment with OC Kappa

*A. thaliana* plants of 15 days of age (*n* = 8 each sample) were sprayed on leaves 5 times with 1 mL every 2 days with water (control) or with an aqueous solution of OC kappa at a concentration of 1 mg mL^−1^ and cultivated for 5 or 15 additional days. Plants (*n* = 4) cultivated for 5 additional days were collected, and the rosette diameter and fresh and dry weight biomass were determined. Plants cultivated for 15 additional (*n* = 4) days were collected, and length of primary root and number of leaves, flowers, and siliques were determined. For total RNA extraction, *A. thaliana* plants of 15 days of age (*n* = 6) were sprayed, once, with water (control) or with an aqueous solution of OC kappa at a concentration of 1 mg mL^−1^ and collected at 0, 6, 12, 24, 48, 72, and 96 h after treatment, then stored at −80 °C.

### 4.3. Total RNA Extraction, Preparation of cDNA Libraries, and Sequencing

Total RNA was isolated from control and treated plants 0, 6, 12, 24, 48, 72, and 96 h after treatment using the EZNA total RNA Kit (Omega Biotek, Norcross, GA, USA). *A. thaliana* plants (100 mg of each sample) were frozen in liquid nitrogen and homogenized in 0.5 mL of RB buffer with 20 µL mL^−1^ of 2-mercaptoethanol. The samples were centrifuged, and the supernatant was recovered and mixed with ethanol 70%. The solution was transferred to the HiBind RNA mini column and washed with RNA Wash Buffer I and II. Total RNA was eluted with 50 μL of DEPC-treated water. Total RNA integrity was evaluated using a fragment analyzer and the software PROsize version 5.0.1.3 (Advanced Analytical Technologies, Orangeburg, SC, USA). RNA samples were sent to Novogene Inc. (Sacramento, CA, USA), and paired-end cDNA libraries were prepared from samples obtained at 0, 6, 12, and 24 h after treatment and sequenced using a Hi-Seq Illumina 4000 (San Diego, CA, USA).

### 4.4. Annotation and Detection of Differentially Expressed Transcripts

Reads obtained by RNA-seq were trimmed using Prinseq (version 0.20.4; −min_len 50 -min_qual_mean 20 -ns_max_n 1 -derep 14 -derep_min 9 -lc_method dust -lc_threshold 49 -trim_left 10 -trim_qual_right), and the quality-controlled reads were visualized in Fastqc [35]. The reads were mapped and classified using OmicsBox software (Barcelone, Spain) [36]. Annotated sequences were classified according to their Gene Ontology [37] using OmicsBox software [36], and those having an e-value of 1 e^−6^ were selected. Sequences were classified according to GO domain (biological process, molecular function, and cellular component) using the functional module of the OmicsBox software. The differentially expressed genes (DEG) were searched and analyzed by the transcriptomics module of the OmicsBox software [36], and DEG were identified and counted and compared using EdgeR (version 3.20.2) [38] at an FDR < 0.005. Differentially expressed transcripts were obtained by contrasting samples at different time points: control 6 vs. treated 6; control 12 vs. treated 12; and control 24 vs. treated 24.

### 4.5. Quantification of Chlorophylls

The levels of chlorophylls were determined as described in Lichtenthaler and Wellburn (1983) [39]. *A. thaliana* leaves (100 mg of FT) were frozen in liquid nitrogen and homogenized with 2 mL 80% acetone. The mixture was centrifuged at 3000 rpm for 10 min at 4 °C, and the supernatant was recovered and diluted 10 times with 80% acetone. The absorbance was determined at 649 and 663 nm. The levels of chlorophylls a and b were calculated according to the formula:

Chlorophyll a (μg mL^−1^) = 12.7 × A663 nm − 2:69 × A645 nm

Chlorophyll b (μg mL^−1^) = 22.9 × A645 nm − 4:68 × A663 nm

### 4.6. Quantification of Carotenoids

The level of carotenoids was determined as described in Lichtenthaler and Wellburn (1983) [39] with modifications. *A. thaliana* leaves (100 mg) were frozen in liquid nitrogen and homogenized in a mortar using a pestle. A total of 10 mL of acetone (80%) was added, then the mixture was shaken using a vortex for 10 s and incubated 15 min in ice. The mixture was centrifuged at 3000 rpm for 10 min at 4 °C, and the supernatant was recovered. The absorbance was determined at 480 and 510 nm in a spectrophotometer (Agilent, model Cary 8454, Santa Clara, CA, USA) and the level of carotenoids was determined using the formula:

Carotenoids (mg g^−1^) = 7.6 × A480 nm × DF × V × (1 cm × W × 1000)^−1^

V = final volume of chlorophyll extract in 80% acetone (mL)

W = fresh weight of leaf tissue (g); DF = Dilution Factor

### 4.7. Quantification of Transcripts of Enzymes Involved in C, N, and S Assimilation by qRT-PCR

cDNA was synthesized from each sample (1 µg of total RNA) with an iScripts Reverse Transcription for RT-qPCR (BioRad, Hercules, CA, USA). qPCR was performed with SsoAdvanced Universal SYBR Green Supermix (BioRad) in an Aria MX real-time PCR thermocycler (Agilent, Santa Clara, CA, USA). Tubuline (AT1G20010.1) was used to normalize transcript level in control and treated conditions [40]. The levels of transcript encoding the small subunit chain 1A of rubisco (AT1G67090.1), glutamine synthase (AT1G48470.1), and O-ASTL (AT2G43750.1) were determined [41]. Sequences of primers used to amplify rubisco, GlnS, and O-ASTL are shown in Supplementary Table S1.

### 4.8. Statistical Analysis

Significant differences in phenotypic analyses were calculated with a two-tail paired *t*-test at a 95% confidence interval followed by a Mann–Whitney post-test. Significant differences in transcript levels, carotenoid levels, and chlorophyll levels were calculated with a one-way ANOVA at a 95% confidence interval followed by a Tukey’s multiple comparison post-test. Analyses were performed using the statistical software Prism 6 (GraphPad Software Inc., CA, USA) and conducted as three independent replicates in triplicate.

## 5. Conclusions

OC kappa stimulates growth in *A. thaliana* plants, resulting in the increase of rosette diameter, FW and DW of plants, and length of the primary root. The increase in growth may be due to an increase in photosynthesis and basal metabolism, mainly in C, N, and S assimilation.

## Figures and Tables

**Table 1:** Up-regulated transcripts involved in growth and development after 12 h of treatment with OC kappa.

Process	ID	Proteins	Log FC
Growth	AT5G03120	MOK16.3	1.8
	AT3G19850	MPN9.9 BTB/POZ	2.1
	AT3G61750	Cyt b561	1.4
	AT4G23820	PECL	1.2
	AT3G28040	MMG15.21	1.1
	AT5G07080	ACT	3.2
Cell growth	AT4G03070	AOP1	1.8
	AT5G24870	UBQT	1.3
	AT5G65380	DTX27	1.7
Unidimensional cell growth	AT5G44020	HAD	1.8
	AT2G37640	EXPA3	1.6
	AT1G69530	EXPA1	2.4
	AT3G29030	EXPA5	1.2
	AT2G40610	EXPA8	6.5
	AT1G75750	GASA1	2.1
	AT2G18300	HBI1	2.8
	AT1G75780	TUBB1	1.3
Pollen tube growth	AT2G43750	OASB	1.6
	AT5G04950	NAS1	2.4
	AT1G09240	NAS3	2.8
	AT5G46220	TOD1	4.3
Meristem growth	AT1G27470	WD-40	1.1
Cell wall modification	AT1G74670	GASA6	2.9
	AT1G48100	PGLR4	2.8
developmental growth	AT3G16175	TES	3.9
	AT1G72880	SurE	1.2
	AT1G04040	HAD	2.8
	AT3G03820	SAUR29	9.7
	AT5G18670	BAM9	2.4
	AT5G65890	ACR1	1.8
	AT1G58520	RXW8	2.5
	AT4G30140	GDSL	2.2
	AT5G24580	HIPPO9	2.9
	AT3G16150	MSL1.19	6.3
	AT3G24480	LRX4	1.2
	AT3G27020	YSL6	1.4
	AT5G48900	PLY20	1.2
	AT1G69160	BIG1E	2.2
	AT3G25717	DVL6	2.4
	AT2G32450	T32F6.3	1.1
	AT5G54130	CBEEP	2.2
	AT4G14740	DL3410C	1.2
	AT2G28470	BGAL8	1.5
	AT1G09250	BHLH149	1.3
Regulation of growth	AT1G26945	PRE6	4.2
	AT5G39860	PRE1	9.2
	AT2G17500	PILS5	1.6
	AT2G26670	HO1	1.4
	AT1G35670	CPK11	1.9
	AT5G64770	GLV2	3.3
	AT1G72430	SAUR78	2.0
Regulation of cell growth	AT3G51800	EBP1	1.5
Regulation of	AT5G15430	CBD	6.7
developmental growth	AT4G25780	CAP	2.0
Response to growth	AT1G10370	GSTU17	4.9
hormone			

**Table 2:** Up-regulated transcripts encoding proteins involved in photosynthesis after 12 h of treatment with OC kappa.

Process	ID	Proteins	Log FC
PSII	AT1G03600	Psb27-H1	1.5
	AT4G28660	Psb28	1.7
	AT2G20890	Psb29	1.1
	ATCG00070	PsbK	1.4
	AT5G66570	PsbO1	1.6
	AT3G50820	PsbO2	2.1
	AT1G06680	PsbP1	2.4
	AT4G15510	PsbP1	2.1
	AT4G21280	PsbQ1	2.9
	AT4G05180	PsbQ2	2.5
	AT1G79040	PsbR	1.8
	AT1G44575	PsbS	2.1
	AT3G21055	PsbT	1.9
	AT2G30570	PsbW	2.2
	AT2G06520	PsbX	2.2
	AT2G06520	PsbX	2.2
	AT1G67740	PsbY	3.2
PSI	AT4G02770	PsaD1	2.5
	AT1G03130	PsaD2	4.0
	AT4G28750	PsaE-1	2.6
	AT2G20260	PsaE-2	1.6
	AT1G31330	PsaF	2.7
	AT1G55670	PsaG	2.5
	AT1G52230	PsaH	3.3
	AT1G30380	PsaK	2.1
	AT4G12800	PsaL	2.3
	AT1G49975	PsaN	1.2
	AT5G64040	PsaN	3.2
	AT1G08380	PsaO	3.1
	AT4G22890	PGRL1A	1.4
LHCII	AT1G29920	LhcB 1.1	4.2
	AT1G29910	LhcB 1.2	3.3
	AT1G29930	LhcB 1.3	3.2
	AT2G34430	LhcB 1.5	1.2
	AT2G34420	LhcB 1.5	2.7
	AT2G05100	LhcB 2.1	3.0
	AT2G05070	LhcB 2.2	3.7
	AT3G27690	LhcB 2.4	3.9
	AT5G54270	LhcB 3	1.8
	AT5G01530	LhcB 4.1	8.0
	AT3G08940	LhcB 4.2	7.7
	AT4G10340	LhcB 5	7.3
	AT1G15820	LhcB 6	4.4
	AT1G76570	LhcB 7	5.3
LHCI	AT3G54890	LHCA 1	7.4
	AT3G61470	LHCA 2	1.9
	AT1G61520	LHCA 3	3.7
	AT4G17600	LIL3.1	1.5
	AT3G47470	LHCA 4	1.6
	AT1G45474	LHCA 5	2.0
	AT1G19150	LHCA 6	1.7
Electron transport chain	AT1G15980	PnsB1	1.6
	AT1G76100	PETE	1.4
	AT5G08720	PPP1	1.9
Chlorophyll synthesis	AT1G74470	CHLP	2.5
	AT3G56940	CRD1	3.4
	AT4G14690	ELIP2	7.1
Biosynthesis, repair and	AT1G16720	Hcf173	2.2
reassembly of PSII	AT4G35250	Hcf244	2.3
	AT1G75690	Lqy1	2.2
	AT5G02120	OHP1	2.5
ATP synthase	AT4G00895	ATP synthase subunit delta, ATPaseF1	1.2
	AT4G09650	ATP synthase subunit delta, ATPaseF1	2.9

**Table 3:** Up-regulated transcripts encoding proteins and enzymes involved in C, N, and S assimilation after 12 h of treatment with OC kappa.

Process	ID	Proteins	Log FC
Calvin–Benson cycle	AT1G10960	FD1	3.0
	AT4G14890	FDC1	1.8
	AT5G66190	FNR1	1.2
	AT4G38970	FBA2	2.0
	AT2G21330	FBA1	2.6
	AT1G12900	G3PDHA2	2.5
	AT1G42970	G3PDHB	1.1
	AT3G26650	G3PDHA1	1.4
	AT4G22890	PGRL1A	1.4
	AT2G01290	R5PI2	2.5
	AT3G04790	R5PI3	2.0
	AT1G67090	RBCS-1A	1.5
	AT5G38430	RBCS-1B	2.5
	AT5G38410	RBCS-3B	2.7
	AT5G38420	RBCS-2B	2.7
	AT2G39730	RBCA	3.0
	AT3G04550	RAF1.2	1.3
	AT5G19855	RBCX2	1.4
	AT3G02730	TRXF1	1.2
	AT1G03680	TRXM1	1.5
	AT3G06730	ClTRX	1.6
	AT1G76080	CDSP32	1.7
	AT5G16400	TRXF2	2.2
	AT5G17630	XPT	1.6
	AT5G46110	TPT	1.9
	AT3G01550	PPT2	4.1
Nitrogen assimilation	AT5G56860	GATA21	1.1
	AT1G66200	GLN1-2	1.8
	AT5G35630	GLN2	1.6
	AT1G52190	NPF1.2	1.3
	AT2G26690	NPF6.2	2.8
	AT1G12110	NPF6.3	2.5
	AT3G21670	NPF6.4	5.9
	AT2G15620	NIR1	1.4
Sulfur uptake	AT1G62180	APR2	2.9
	AT4G21990	APR3	1.8
	AT5G67520	APK4	3.4
	AT2G43750	OASB	1.6
	AT5G28030	DES1	1.9
	AT5G28020	CYSD2	3.2
	AT2G25680	SULTR2	2.5
	AT3G51895	SULTR3	1.7
	AT1G61740	TAUE2	1.0
	AT2G36630	TAUE2	2.6

## Data Availability

Data are available in the NCBI database as Bioproject PRJNA980612.

## Supplementary Materials

The following supporting information can be downloaded at: https://www.mdpi.com/article/10.3390/ijms241511894/s1.
