# Supplementary material for: Oligo-Carrageenan Kappa Increases Expression of Genes Encoding Proteins Involved in Photosynthesis, C, N, and S Assimilation, and Growth in Arabidopsis thaliana
Source: Int J Mol Sci. 2023 Jul 25;24(15):11894. doi: 10.3390/ijms241511894 (PMC10418774; doi:10.3390/ijms241511894)
Supplement: Supplementary file 1 [file ijms-24-11894-s001.zip › supplemetary Figure S1.pdf]

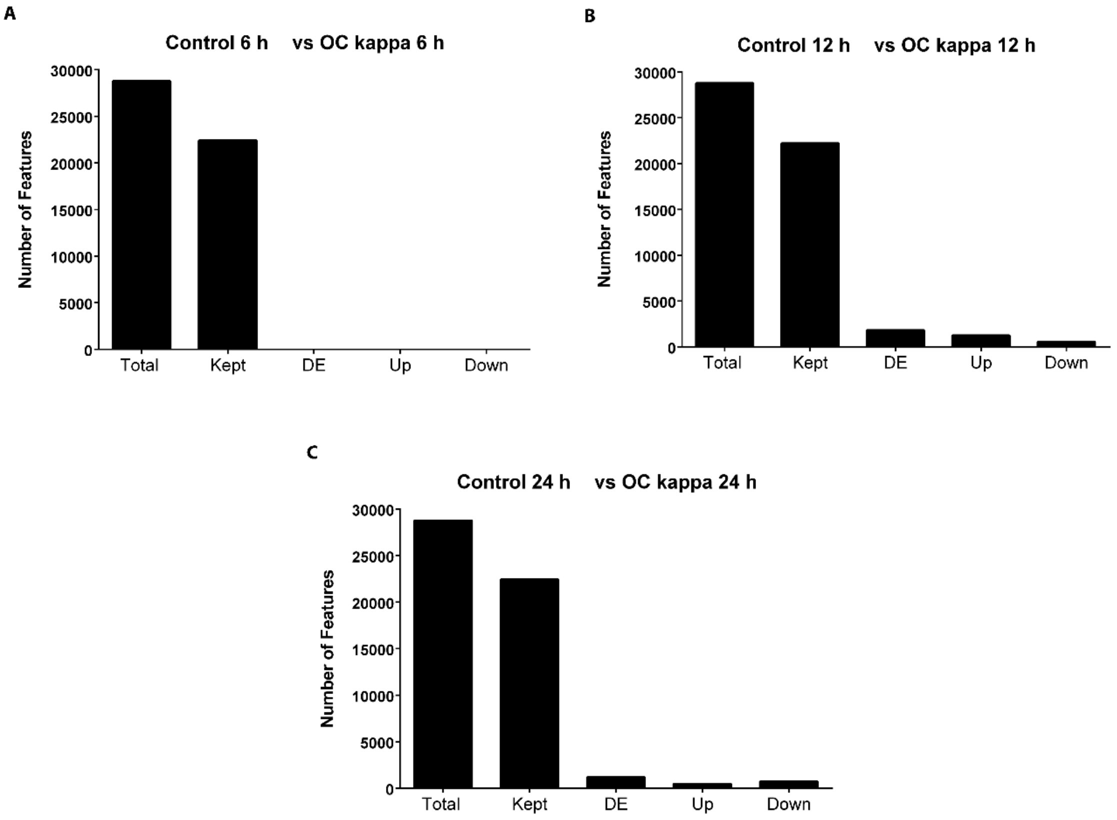

Scheme S1. Number of total expressed transcripts (total), number of non-redundant transcripts (kept) in treated plants and number differentially expressed transcripts (DE), up-regulated (Up) and down-regulated (Down) transcripts in plants treated with OC kappa at 1 mg mL^{-1}, once, and cultivated for 6 h (A), 12 h (B) and 24 h (C) compared to control plants at the same time.
